# Willingness-to-pay and willingness-to-accept of informal caregivers of dependent elderly people in Shanghai, China

**DOI:** 10.1186/s12913-020-05481-2

**Published:** 2020-07-06

**Authors:** Wenwei Liu, Tongzhou Lyu, Xiaoyi Zhang, Suwei Yuan, Huimin Zhang

**Affiliations:** 1 College of Philosophy, Law and Political Science, Shanghai Normal University, Shanghai, China; 2 School of International and Public Affairs, Shanghai Jiao Tong University, Xin Jian Building, 1954 Huashan Rd, Shanghai, 200030 China; 3 China Hospital Development Institute, Shanghai Jiao Tong University, Shanghai, China

**Keywords:** Informal care, Economic valuation, Contingent valuation, Informal caregiver

## Abstract

**Background:**

In order to make optimal long-term care-related decisions, it is important to take a societal perspective. Shanghai is one of the pilot cities of social long-term care insurance in China. However, little knowledge exists about the economic value of the informal care provided to dependent elderly people in China. This paper aims to evaluate the economic value of informal caregiving in Shanghai using the contingent valuation method by their least-preferred care tasks, and identify the associated factors of willingness-to-pay (WTP) and willingness-to-accept (WTA) of the informal caregivers.

**Methods:**

This study employed the contingent valuation method to elicit 371 informal caregivers’ WTP and WTA for 1 hour of reduction or increase of least-preferred caring tasks in Shanghai. Univariate and multivariate analyses were conducted to explore the associated factors with the WTP and WTA values.

**Results:**

The average WTP and WTA were 25.31 CNY and 38.66 CNY, respectively. The associated factors with WTP include caregiver’s income and caregiver’s relationship to the recipient. Care recipient’s age, income, least-preferred task by the caregiver, and subscales of Caregiver Reaction Assessment were found to be associated with WTA. The non-responsiveness rates were 26.1 and 33.2% for WTP and WTA questions, respectively.

**Conclusions:**

The findings of the current study demonstrated that decision-makers and researchers should take the economic valuation results of informal care into account to make more informed and effective long-term care-related policies and analyses.

## Background

With the rapid expansion in China of the aging population, changing morbidity, and evolving lifestyles, the number of elderly people who require long-term care has been growing dramatically [1]. To deal with the “grow old before getting rich” situation [2], in 2016, the government initiated a pilot program of long-term care insurance in 15 cities, one of which is Shanghai.

Studies have demonstrated that a substantial part of long-term care is provided by informal caregivers in China [3, 4]. Informal caregivers refer to non-professionals who provide long-term care services to their relatives, neighbors, friends, etc. [5]. Although unpaid, informal caregiving is not without costs. For example, informal caregivers are reported to devote less time to paid work and leisure [6, 7], have greater morbidity and mortality risks [8, 9], and be confronted with higher levels of stress [10]. In addition, most researchers agree that informal caregiving substitutes and/or complements formal caregiving services, and should be recognized accordingly in long-term care related researches and policies [11, 12].

Consequently, many informal caregiving economic valuation investigations have been carried out in different countries and regions. The results revealed that different valuation methods exert impacts on monetary value. The most utilized evaluation methods are the opportunity cost method, the proxy good method, the contingent valuation method, and the conjoint method [13]. The former two methods are termed *revealed preference method*s, and use market prices of labor to evaluate the input or output of caregiving, respectively. However, both the opportunity cost method and the proxy method have been criticized because both methods rely on the accuracy of caring time recall, fail to reflect the full impact of caregiving, and cannot capture the preference of the caregivers [5]. A previous study also suggests that the revealed preference method is less applicable in certain contexts, especially in less developed countries, because most caregivers are without paid work [14]. The contingent valuation method (CVM) and the conjoint method constitute *stated preference methods*. Among stated preference methods, the CVM is one of the most utilized, because it is less cognitively demanding and more sensitive to the heterogeneity of caregiving [15]. CVM is generally conducted by asking the amount of money that the respondent is willing-to-pay (WTP) or willing-to-accept (WTA) for a reduction or increase of a unit of caregiving time. Moreover, associated factors, differences, and non-responsiveness of WTP and WTA were examined. The results differ according to study design, sample characteristics, countries, regions, etc. Generally, WTP and WTA are associated with income and/or wealth, health status, and care burdens on caregivers [16]. Most CVM studies of informal care were conducted in developed countries with well-established long-term care service systems. However, few such researches were performed in developing countries, and none has been found in China.

Due to Asian filial norms, the developing social insurance system, and scarcity of care resources, informal caregiving has always been the most important source of long-term care in China [17, 18]. However, many scholars have pointed out that the rapidly growing elderly population, one-child policy, and increasing female labor participation could jointly affect the traditional way of growing old for Chinese people, and the demand for formal care is predicted to rise markedly [19, 20]. Shanghai has the largest population of elderly people among all cities in China. In 2016, Shanghai was selected as one of the first piloting cities of the social long-term care insurance. Informal caregivers of the insured elderly choose the service type from a list provided by the government, and then the insurance will co-pay for the chosen services provided by designated providers. The out-of-pocket expense is fixed, irrespective what services are utilized. Moreover, the needed hours of care to be co-paid by LTC insurance is determined by a third-party assessment institution, and the assessment result for an elderly individual is usually a few hours per week (from 1 to 7 h) due to the serious shortage in supply of home care [21]. The reassessment of demand is conducted every 2 years, unless the applicant requests for a reassessment. This ‘managed demand’ strategy can avoid possible rapidly-growing costs of home care, alleviate the burdens of informal caregivers as well as emphasize the responsibility of both the family and the government.

Few investigations have explored burdens of informal care providers in China. Studies concerning informal care mostly focused on caring time, depression risk, and the burden of informal caregiving [6, 22]. One study utilized open access data to evaluate the economic value of informal care provided to stroke survivors using the opportunity cost method [23]. However, as previously mentioned, revealed preference methods fail to represent all costs involved in caregiving, especially in developing countries. At present, there is no extant literature on the economic valuation of informal care by CVM in China. However, since the population is aging rapidly and the long-term care service system in China is being overhauled, it is of pivotal importance to elucidate the economic valuation of informal care in China. Therefore, this paper aims to evaluate the economic value of informal caregiving in Shanghai using the contingent valuation method (CVM) by the least-preferred care tasks, and identify the associated factors of WTP and WTA of caregivers.

## Methods

### Sample and study design

A total of 439 caregivers were interviewed. Caregivers were recruited through 31 randomly-selected communities in Shanghai. All of the informal caregivers of elderly people applying for social long-term insurance from January 1, 2019, to June 1, 2019 at the Neighborhood Committees of the community were invited to participate. The caregivers must be 16-years-old or older, and have spent at least 1 hour per day performing care tasks for the applicant. Written and verbal consent of participants in the economic valuation project were obtained prior to the interview. On average, the interview took 30 min to complete.

### Economic valuation methods

Questionnaire-based face-to-face interviews were conducted by five trained interviewers. The questionnaire was developed for this study, and has not been published elsewhere (see Additional file 1). In order to avoid over-pledging bias, which was observed in other CVM studies [24], the caregivers were interviewed independently without the presence of their care recipients.

Contingent values of WTP and WTA were obtained via open-ended questions. The approach of open-ended questions in CVM studies was controversial in valuing environmental improvements and pollutions [25]. However, it has been successfully employed in several studies valuing informal care in developed countries and regions [16, 26, 27], and empirical evidence shows that this approach can also be employed in low-income countries [14, 28]. Before the CVM questions were proposed, the respondents were first asked to identify which is their least-preferred task among all of the care tasks when taking care of the recipient. Many CVM studies have posed contingent questions in general, for example, by asking what is the maximum/minimum amount of money that the caregiver would be willing to pay/accept for a 1 hour increase/decrease of caring, without specifying the care task. However, considering that one of the main aims of this research is to provide solid empirical evidence for informed long-term care related policy decisions, and the least-preferred task is the service that is most likely to be “contracted out” to the services market and co-paid by the long-term care insurance, especially when the demand for care is managed, the research team decided to obtain WTP and WTA according to the least-preferred tasks. In case of non-responsiveness of the caregivers because of discomfort with sacrificing the benefits of the recipients, we informed the interviewees that the government will send qualified professional caregivers to take care of the recipient. This method has been successfully utilized in several researches [5, 14, 16]. Previous investigations also employed a hypothetical scenario, in which the reduction of caregiving is because the care recipient’s health had improved, or there is a hypothetical recipient [29]. In the pilot study conducted with a small sample of 30 care recipients, these two scenarios were included. However, the improvement of recipients’ health scenario is more cognitively demanding, because most elderly people presented with progressive diseases, in which the passing of time generally leads to deterioration. Moreover, caregivers can also refuse to provide an answer if they do not want to reduce/increase 1 hour of caring per day. The scenario of a hypothetical care recipient was also not adopted, because it is difficult for the real caregivers to imagine taking care of a stranger in WTP questions, and this study only aims to value the informal care provided to real recipients. Examples of the WTP and WTA questions posed are as follows, respectively:

*Please imagine that the government is to send professional care workers to provide services. What is the maximum amount of money that you are willing to pay for an hour of reduction in caregiving of your least-preferred care task per week?*

*Please imagine that the government is going to subsidize the care that you are currently providing to your recipient. How much is the minimum sum that you are willing to accept for this extra hour of your least-preferred care task per week?*

### Data and statistical methods

Socio-demographic, socio-economic, and health-related data were collected for the analysis of factors associated with WTP and WTA. Age, gender, education, income (per month), and residential information (co-residence) of the caregiver and the recipients were collected. In addition, questions about employment, marital status, and relationship to the recipient of the caregivers were also asked during the interviews. Instead of subjective measures of the health status of the recipients, the Charlson Comorbidity Index (CCI) was employed as an indicator of the health status of the recipients. CCI is an index that assigns each morbidity a score ranging from 1 to 6 according to its severity and resource-intensity [30], in which a higher CCI indicates a worse health status.

Since WTP and WTA were also found to be associated with various care-related factors, the objective and subjective burden of caring, and the availability of other source of care (informal or formal) were also analyzed. The objective burden of caring was measured by care duration and care time spent on caring per week. Care duration was assessed by asking how long the respondent has been taking care of the care recipient. Time spent on caring was measured via 17 tasks, including household activities of daily living tasks (HDL), activities of daily living (ADL) tasks, instrumental ADL (IADL), and supervision and companion time, which is in accordance with the extant literature to provide comparable results [5, 16, 31]. The weekly recall method was adopted for recording the time spent on each task. Respondents were asked how much time they spent on each of the 17 tasks in the previous week. Considering the difficulty in discerning exaggeration in care hours by care tasks, if the total care time exceeded 256 h per week (16 h per day), the questionnaire would be considered as invalid. The subjective burden of caring was assessed using the Caregiver Reaction Assessment (CRA) [32]. There are five subscales of the CRA, each of which measures a dimension by a battery of questions. These dimensions include disrupted schedule, self-esteem, lack of family support, loss of physical strength, and financial problems. The scales were from 1 to 5, in which a higher score indicates a higher subjective burden, except for the subscale of self-esteem, which was coded inversely. Since this study is interested in the impact of specific subjective sources of burden on WTP and WTA, overall subjective burden was not assessed. The availability of other sources of caring were obtained by asking the respondents whether any other people were assisting with taking care of the care recipient, either paid or unpaid.

Descriptive data of continuous variables and categorical variables were presented by means and standard deviations (SDs), and number and percentage, respectively. Univariate and multivariate analysis were performed to explore factors associated with higher WTP and WTA of the caregivers. Student’s *t*-test, one-way analysis of variance (ANOVA), Chi-square test, and Spearman’s rank correlation test were employed according to the nature of the variables in the univariate analysis. Variables significantly associated with WTP and WTA in the univariate analysis were introduced to the multivariate analysis. The stepwise ordinary least square (OLS) regression method was utilized for the multivariate analysis. A *p* value < 0.200 was considered statistically significant in the univariate analyses to avoid confounding factors [33], and a *p* value < 0.050 was adopted for the multivariate analysis. Microsoft Office Excel version 2010 and SPSS version 20.0 were used for data entry and analysis, respectively.

## Results

### Characteristics of informal caregivers

Among the 439 interviewees, 371 were included in our analysis. Sixty-eight (68) were excluded due to incomplete information (*N* = 6), under 16-years-old (*N* = 7), and over 16 h of caring per day (*N* = 55). The characteristics of the informal caregivers are presented in Table 1. Female caregivers constituted 58.2% (*N* = 218) of the sample. The average age of the caregivers was 56.67 (SD = 11.77). Most of the caregivers were married (*N* = 305, 91.6%) and had 7-to-15 years of education (*N* = 213, 64.4%). Employed, retired, and unemployed accounted for 48.2% (*N* = 179), 28.6% (*N* = 106), and 23.2% (*N* = 86), respectively. 9.3% (*N* = 123) of the primary caregivers had a monthly income of less than 4000 CNY, 44.4% (*N* = 139) from 4000 to 6000 CNY, and 51 (16.3%) above 6000 CNY. In addition, a majority of the caregivers were children of the recipients (*N* = 227, 67.0%), and 26.3% (*N* = 89) of the primary caregivers were spouses. 3 47.7% (*N* = 165) of the respondents were living with the recipients. Care recipients were mostly women (*N* = 238, 64.9%), and the average age was 80.73 (SD = 8.76). Most care recipients had 7-to-15 years of education (*N* = 203, 57.0%), and had a monthly income below 4000 CNY (*N* = 184, 50.3%). 19.2% (*N* = 70) of the elderly people had a CCI equal to zero. 67.1% (*N* = 245) and 13.7% (*N* = 50) of the recipients’ CCI were 1 to 2 and above 2, respectively. On average, the caregivers had been taking care of the recipients for 7.44 years (SD = 4.82), and the mean time of caring was 42.89 h (SD = 30.15) per week. Specifically, on average, the caregivers spent 13.19 (SD = 10.76), 8.42 (SD = 7.80), 10.04 (SD = 9.76), and 11.08 (SD = 13.05) hours per week on HDL tasks, ADL tasks, IADL tasks, and supervision and companionship, respectively. Mean scores of subjective burden measured by the CRA were 2.77 (SD = 0.53), 2.64 (SD = 0.69), 2.67 (SD = 0.68), 1.14 (SD = 0.46), and 1.36 (SD = 0.71) for disrupted schedule, self-esteem, family support, physical strength, and financial issue, respectively. 37.7% (*N* = 140) of the recipients received informal care from caregivers other than the primary caregiver, and 55.8% (*N* = 207) of the recipients were also using formal care.

**Table 1:** Characteristics of the caregivers and care recipients

	N/Mean	%/SD
**Characteristics of the caregivers**		
Gender		
Male	153	41.2
Female	218	58.2
Age	56.67	11.77
Marital status	(missing = 38)	
Married	305	91.6
Widowed	12	3.6
All others	16	4.8
Education	(missing = 40)	
0–6 years	14	4.2
7–15 years	213	64.4
Above 15 years	104	31.4
Employment		
Employed	179	48.2
Retired	106	28.6
Unemployed	86	23.2
Income (CNY)	(missing = 58)	
< 4000	123	39.3
4000–6000	139	44.4
> 6000	51	16.3
Relationship to the recipient	(missing = 32)	
Spouse	89	26.3
Child	227	67.0
All others	23	6.8
Co-residence	(missing = 25)	
Yes	165	47.7
No	181	52.3
**Characteristics of the care recipients**		
Gender	(missing = 4)	
Male	129	35.1
Female	238	64.9
Age^a^	80.73	(8.76)
Education	(missing = 15)	
0–6 years	94	26.4
7–15 years	203	57.0
Above 15 years	59	16.6
Income (CNY)	(missing = 5)	
< 4000	184	50.3
4000–6000	149	40.7
> 6000	33	9.0
CCI	(missing = 6)	
CCI = 0	70	19.2
CCI < =2	245	67.1
CCI > =3	50	13.7
**Caregiving-related measures**		
Care duration (years)^a^	7.44	4.82
Care time (hours/week)^a^	42.89	30.15
HDL tasks	13.19	10.76
ADL tasks	8.42	7.80
IADL tasks	10.04	9.76
Supervision and companionship	11.08	13.05
CRA^a^	(missing = 18)	
CRA – Disrupted schedule	2.64	.71
CRA – Self-esteem	2.77	.53
CRA – Lack of family support	2.63	.69
CRA – Loss of physical strength	2.67	.68
CRA – Financial issue	1.16	.50
Receives formal care		
Yes	207	55.8
No	164	44.2
Receives other informal care		
Yes	140	37.7
No	231	62.3

^a^Mean and SD

### WTP and WTA of informal caregivers

The average maximum WTP and minimum WTA of the respondents were 25.31 CNY (SD = 18.10) and 38.66 CNY (SD = 22.95) for 1 hour of decreasing and increasing in least preferred informal care task per week, respectively (see Table 2). Most caregivers found ADL (*N* = 126, 38.7%) and IADL tasks (*N* = 104, 31.9%) to be the least-preferred tasks. The average WTP for ADL and IADL tasks were 26.11 CNY (SD = 19.33) and 22.28 CNY (SD = 14.32) for 1 hour of reduction in caring, respectively. The average WTA of ADL and IADL tasks were 36.58 CNY (SD = 23.19) and 32.07 CNY (SD = 15.36) for one additional hour of the least-preferred task, respectively. Although the least frequent to be identified as the least-preferred task (*N* = 17, 5.2%), the highest WTP and WTA were found in supervision and companionship tasks, which were 31.25 CNY (SD = 20.35) and 50.00 CNY (SD = 36.33), respectively. Moreover, significant differences of WTP and WTA were found in all four categories of care tasks (*p* < 0.001), which means, on average, the respondents required significantly higher compensation for one extra hour of the least-preferred task than their WTP for 1 hour of decrease of the same task. There were 3 and 1 respondents who provided us with zero values in WTP and WTA, respectively.

**Table 2:** WTP and WTA of caregivers according to least-preferred care tasks

Least-preferred task	N (%)	Mean WTP (SD)	Mean WTA (SD)	*p*
HDL	79 (24.2)	27.59 (19.77)	46.34 (24.55)	< 0.001
ADL	126 (38.7)	26.11 (19.33)	36.58 (23.19)	< 0.001
IADL	104 (31.9)	22.28 (14.32)	32.07 (15.36)	< 0.001
Supervision and companionship	17 (5.2)	31.25 (20.35)	50.00 (36.33)	< 0.001
Overall	326 (100)	25.31 (18.10)	38.66 (22.95)	< 0.001

### Factors associated with economic valuation of informal care giving

Univariate analyses and multi-variate analyses were conducted to identify factors associated with economic valuation of the caregivers. A lower WTP was associated with an older age of the caregiver (Spearman’s correlation = − 0.134, *p* = 0.032) (see Table 3). Significant differences of WTP were also observed in different education (*p* = 0.036), employment (*p* = 0.038), income (*p* = 0.004), relationship to the recipient (*p* = 0.002), and co-residence groups of the caregivers (*p* = 0.022). Regarding the characteristics of the recipient, WTP was significantly correlated with age (Spearman’s correlation = − 0.082, *p* = 0.200), income (*p* = 0.063), and CCI (*p* = 0.198). No caregiving-related variables were found to be significantly associated with WTP. Relationship between the caregiver and the recipient (*p* = 0.168), age (Spearman’s correlation = − 0.178, *p* = 0.007), income (*p* = 0.014), and CCI (*p* = 0.134) of the recipient were determined to be significantly correlated with WTA of the caregivers. WTA was also significantly associated with several caregiving-related variables, including care time in HDL tasks (Spearman’s correlation = 0.113, *p* = 0.082), least-preferred care tasks (*p* = 0.001), CRA scores of disrupted schedule (Spearman’s correlation = 0.105, *p* = 0.106), lack of family support (Spearman’s correlation = − 0.127, *p* = 0.046), and loss of physical strength (Spearman’s correlation = 0.133, *p* = 0.036).

**Table 3:** Univariate analysis of factors associated with WTP and WTA

	Mean WTP (SD)	*p*	WTA (SD)	*p*
**Characteristics of caregivers**				
Gender				
Male	26.17 (19.14)	0.482	39.98 (26.23)	0.408
Female	24.62 (17.27)		37.55 (19.83)	
Age^a^	−0.134	0.032	−.036	0.587
Marital status				
Married	25.61 (18.44)	0.741	38.77 (23.05)	0.700
Widowed	24.06 (14.93)		53.33 (26.58)	
All others	21.45 (18.13)		38.75 (22.99)	
Education				
0–6 years	22.63 (18.52)	0.036	41.86 (24.51)	0.223
7–15 years	23.46 (15.27)		36.72 (21.35)	
Above 15 years	29.53 (21.32)		42.55 (29.09)	
Employment				
Employed	26.50 (19.41)	0.038	40.03 (25.37)	0.581
Retired	20.71 (14.74)		37.41 (24.06)	
Unemployed	25.31 (17.39)		36.21 (20.30)	
Income (CNY)				
Below 4000	21.21 (17.92)	0.004	37.29 (22,17)	0.249
4000–6000	27.10 (16.54)		40.34 (24.63)	
Above 6000	31.59 (22.48)		44.83 (27.19)	
Relationship to the recipient				
Spouse	21.49 (15.50)	0.002	43.17 (27.34)	0.168
Child	25.72 (18.13)		37.92 (23.17)	
All others	39.87 (23.33)		30.00 (16.83)	
Co-residence				
Yes	22.69 (15.11)	0.022	40.71 (25.66)	0.443
No	27.73 (19.76)		38.26 (22.91)	
**Characteristics of care recipients**				
Gender				
Male	23.49 (19.37)	0.242	39.24 (28.27)	0.733
Female	26.22 (17.47)		38.13 (21.85)	
Age^a^	−0.082	0.200	−0.178	0.007
Education				
0–6 years	24.03 (16.31)	0.416	34.84 (22.50)	0.387
7–15 years	25.22 (19.26)		39.60 (26.20)	
15 years and above	28.62 (18.58)		42.35 (19.82)	
Income (CNY)				
Below 4000	22.80 (15.81)	0.063	34.22 (21.67)	0.014
4000–6000	28.12 (20.86)		43.01 (26.10)	
Above 6000	26.70 (15.11)		43.27 (20.27)	
CCI				
CCI = 0	27.06 (14.38)	0.198	35.94 (18.05)	0.134
0 < CCI < =2	25.84 (20.38)		40.84 (27.67)	
CCI > =3	20.91 (12.40)		33.27 (15.95)	
**Caregiving-related measures**				
Care duration (years)^a^	0.052	0.399	−0.006	0.929
Care time (hours/week)				
HDL tasks	− 0.073	0.235	0.113	0.082
ADL tasks	0.033	0.586	−0.039	0.549
IADL tasks	0.063	0.306	−0.042	0.512
Supervision and companionship	−0.019	0.758	−0.063	0.322
Least-preferred care task				
HDL	27.59 (19.77)	0.222	46.34 (24.55)	0.001
ADL	26.11 (19.33)		36.58 (23.19)	
IADL	22.28 (14.32)		32.07 (15.36)	
Supervision and companionship	31.25 (18.27)		50.00 (36.33)	
CRA				
CRA – Disrupted schedule	−0.034	0.588	0.105	0.106
CRA – Self-esteem	−0.001	0.985	0.022	0.728
CRA – Lack of family support	−0.002	0.968	−0.127	0.046
CRA – Loss of physical strength	0.034	0.572	0.133	0.036
CRA – Financial problem	−0.049	0.421	−0.033	0.604
Formal care				
Yes	26.13 (19.24)	0.339	39.09 (23.18)	0.716
No	23.97 (16.06)		37.93 (25.80)	
Other informal care				
Yes	25.58 (20.18)	0.830	40.57 (28.56)	0.262
No	25.11 (16.50)		37.11 (19.82)	

^a^Spearman correlation

The results of the multivariate analysis are presented in Table 4. Lower caregiver income – below 4000 CNY (*β* coefficient = − 6.145, 95% CI *β* coefficient = − 10.404 – − 1.886, *p* = 0.005), being the spouse (*β* coefficient = − 22.980, 95% CI *β* coefficient = − 33.264 – − 12.696, *p* < 0.001) or a child (*β* coefficient = − 16.555, 95% CI *β* coefficient = − 26.289 – − 6.821, *p* = 0.001), and older care-recipient’s age (*β* coefficient = 0.302, 95% CI *β* coefficient = − 0.530 – − 0.075, *p* = 0.010) were significantly associated with lower WTP values. Concerning WTA, higher values were correlated with the least-preferred task being HDL (*β* coefficient = 13.372, 95% CI *β* coefficient = 7.434–19.310, *p* < 0.001) and higher CRA score of disrupted schedule (*β* coefficient = 5.509, 95% CI *β* coefficient = 1.621–9.398, *p* = 0.006). Lower values of WTA were associated with the recipient’s age (*β* coefficient = − 0.619, 95% CI *β* coefficient = − 0.928 – − 0.309, *p* < 0.001), lower care-recipient’s income – below 4000 CNY (*β* coefficient = − 6.109, 95% CI *β* coefficient = − 11.564 – − 0.653, *p* = 0.028), and higher subjective burden of lacking family support (*β* coefficient = − 9.444, 95% CI *β* coefficient = − 13.502 – − 5.386, *p* < 0.001).

**Table 4:** Multivariate analysis of associated factor with WTP and WTA

	*β* coefficient	95% CI *β* coefficient	SE	Standardized *β* coefficient	*p*
**WTP model (*R***^***2***^ **= 0.156, adjusted*R***^***2***^ **= 0.138,*p*** **< .001)**					
(Constant)	67.689	47.140–88.239	10.417		< 0.001
Caregiver’s income - below 4000	−6.145	−10.404 – −1.886	2.159	−.0192	0.005
Relationship to the recipient - spouse	−22.980	−33.264 – −12.696	5.213	−0.663	< 0.001
Relationship to the recipient - child	−16.555	−26.289 – − 6.821	4.935	− 0.501	0.001
Care recipient’s age	−0.302	−0.530 – − 0.075	0.115	−0.188	0.010
**WTA model (*R***^***2***^ **= 0.230, adjusted*R***^***2***^ **= 0.209,*p*** **< .001)**					
(Constant)	96.565	63.343–127.788	15.829		< 0.001
Care recipient’s age	−0.619	−0.928 - -0.309	0.157	−0.271	< 0.001
Care recipient’s income – below 4000	−6.109	−11.564 - -.653	2.766	−0.144	0.028
Least-preferred task – HDL	13.372	7.434–19.310	3.011	0.285	< 0.001
CRA – disrupted schedule	5.509	1.621–9.398	1.972	0.185	0.006
CRA – lack of family support	−9.444	−13.502 – −5.386	2.057	0.323	< 0.001

### Non-responsiveness of WTP and WTA

A major drawback of CVM is the cognitive burden on the caregiver to state a price on caregiving, resulting in high non-responsiveness [15, 34]. Indeed, 12.1% (*N* = 45) of the respondents did not reveal the least-preferred care task. The rates of non-responsiveness of WTP and WTA were 26.1% (*N* = 97) and 33.2% (*N* = 123), respectively. 86 (23.2%) of the respondents did not provide both WTP and WTA. Specifically, 13 (3.5%) of the respondents did not state a WTP, and 37 (10.0%) declined to answer the WTA question.

## Discussion

On average, informal caregivers in Shanghai provide approximately 42.89 h per week of caring. This result is substantially higher than that found in similar researches conducted in The Netherlands (27.1 h per week) [5], Thailand (23.7 h per week) [35], the U.S. (17.9 h per week) [36], and India (38.6 h per week) [37]. Although these studies were targeted to measure recipients with varied characteristics, for instance, patients with different illnesses, the large disparity calls for attention. Moreover, since informal caring constitutes the major source of long-term care for the elderly in China [3], it is critical to understand the care burden experienced by these caregivers. According to the results of the current study, we strongly recommend researchers and decision-makers to include all costs and effects, both direct and indirect, in cost-benefit analysis in studies and policy evaluation projects when it involves a potential gain or loss of benefits of informal caregivers. In addition, we assert that CVM can be employed as a technique for valuating informal care in China, since it produces results which are sensitive to the characteristics of caregivers and recipients, and care tasks.

The WTP and WTA of a reduction or an increase of care per week were 25.31 CNY and 38.66 CNY, respectively, which are lower than the price of formal home care in Shanghai, which was 65 CNY, according to informal interviews with several social workers in neighborhood committees. This phenomenon was also observed in several previous investigations [5, 16, 38]. This difference could constitute evidence that providing care can also produce positive effects on caregivers’ utility [39, 40]. Our research also differentiated four categories of tasks, and employed CVM for the least-preferred one. CVM has been criticized for its inability to capture heterogeneity of unobserved preferences [38]. Most previous studies employed general questions by asking the WTP and/or WTA of a reduction or an increase in caring per week without specifying care tasks. Our findings indicated that caregivers had significantly different WTP and WTA, according to care tasks. ADL tasks were the most frequently mentioned least-preferred care task (38.7%). Controlling for other variables, WTP values were not sensitive to specific care task, but WTA values were significantly higher for caregivers whose least-preferred task was HDL.

Disparities between WTP and WTA were also investigated in this study. In our results, the disparities between WTP and WTA were significant for all four care tasks. A higher WTA was observed in numerous other studies [14, 16]. This disparity between WTP and WTA has several underlying explanations. First, it is possible that caregivers did not include process-utility when deciding on the WTA [40]. Second, these caregivers were reluctant, or it was impossible, to reduce their caring time [26, 41]. Third, the disparity could be ascribed to the income effect, in which WTA does not induce a loss in wealth, and no perfect substitution exists [38, 42]. Moreover, studies with different emphases adopted WTP or WTA accordingly. For example, one study concluded that WTA is more appropriate because informal caregivers constitute suppliers in this “market” [5]. In contrast, a study carried out in Malawi recommended WTP because the answers are more realistic when a loss, rather than a gain, is considered [14].

Lower WTP was associated with lower income of the caregiver, caregiver being the spouse or child, and older age of the recipient. Income was the most common factor that was found to be correlated with WTP in almost all of the extant literature [14, 16, 26, 29]. However, relationship to the recipient was not usually an associated factor in economic evaluation investigations. An underlying reason for the lower WTP of the spouses and children, as well as of caregivers taking care for older recipients, is the strong sense of obligation that they feel to provide care, and thus they were reluctant to reduce 1 hour of caring [43].

Lower WTA was determined to be correlated with older age and lower income of the recipient. Former researches have demonstrated that WTA of the caregiver decreases with the recipient’s health condition and inferior socio-economic status [5, 16, 27]. Higher WTA was determined to be associated with the least-preferred task being HDL, lower CRA score for disrupted schedule, and higher CRA score for lacking family support. Increasing 1 hour of domestic help was not a frequent factor associated with higher WTA. Since household work is the most common care task to be “contracted out” to the market in China, its inclusion in economic thinking “makes more sense” with the respondents accepting compensation for caring [26]. However, to test this hypothesis, further investigation is required. According to the results of the multivariate analysis, in general, WTP is associated with the characteristics and relationship of the caregiver and recipient, while WTA seemed to be more sensitive to caring-related variables. This finding may indicate that, when considering payment for formal care, recipients’ decisions seemed to depend on the status of the caregivers and the recipients. However, regarding WTA and a payment from a third party, the caregivers were likely to make judgements based primarily on the caring activities and the recipient per se. However, these statements necessitate further validation.

In previous empirical studies, CVM was found to possess certain problems, such as non-responsiveness, protest answers, anchoring effects, etc. [26, 44–46]. Compared to other studies using CVM, the non-responsiveness of the WTP and WTA question in the present investigation is relatively high [5]. Moreover, previous researches have shown that informal caregivers were reluctant to reveal their WTA because it is difficult to make monetary estimations for taking care of their loved ones. This explains the significant higher non-responsiveness of WTA than that of WTP that was observed in our interviews. It has also been argued that WTP questions are insensitive in requiring people to state an amount of money they would want to avoid taking care of the recipients. However, since our respondents were caregivers of recipients applying for long-term care insurance, it is reasonable that the responsiveness to WTP questions was greater than that to WTA questions. Indeed, we employed open-ended questions to elicit the WTP and WTA of caregivers to avoid starting-point bias and the anchoring effect, which were observed in other CVM investigations [38]. Researchers have demonstrated that dichotomous questions and bidding games can induce starting-point bias [5, 16, 27]. Considering the explorative nature of this research, and that non-responsiveness is one of the results in which we were interested, no dichotomous, choice, or bid game was followed by CVM questions. Future studies should consider such techniques to avoid high non-responsiveness of CVM studies conducted in China. Considering the very limited number of zero values in this research, we did not use any technique to detect protest answers. However, since protest answers can considerably affect the validity of results, we recommend that future researches employ follow-up questions, which was reported to be useful in a valuation project conducted in Spain [27], to differentiate ‘protest zeros’ and ‘true zeros’.

There are some limitations of this study regarding sampling and measurements of care time. First, our sample was caregivers of recipients who were applying for social long-term care insurance in Shanghai. This may lead to bias because caregivers of non-applicants were not included. However, according to the application policy, all elderly residents in Shanghai who are 60-years-old or older are entitled to apply, irrespective of actual dependency. Second, a considerable percentage of the informal caregivers were retirees or unemployed. Consequently, the use of the CVM could be biased. However, we assumed that the caregivers decided not to join the labor market because the expected wages were at least not higher than the perceived monetary value of the informal care provided. Third, bias could exist in the measure of informal caring time due to employment of the recall method [47]. We attempted to alleviate this impact by a time-specific method. For example, instead of asking “How many hours did you spend on caregiving last week?”, the interviewers asked “From what time to what time did you perform household work for the recipient”, as well as additional questions, such as “How frequently did you take the recipient outside of the house last week, and how long did it take?” to assist the interviewees to recall accurately. Consequently, future studies should consider using the diary method to produce more robust results of care time. Finally, only the monetary values of the least-preferred tasks were estimated in this study, and it should not be interpreted as the average benefits received by informal caregivers by care tasks. To determine this, interested researchers could consider employing the conjoint valuation method or use CVM for each care task to reach this end.

## Conclusions

This paper employed CVM to elicit the WTP and WTA of 371 informal caregivers of dependent elderly applicants for social long-term care insurance in Shanghai, China. The results constituted the first empirical evidence of economic valuation by using CVM of informal care in China, and presented comparable results for international informal care economic valuation studies. The average WTP and WTA of the respondents were 25.31 CNY and 38.66 CNY, respectively. The main factors associated with higher WTP and WTA, and non-responsiveness, were explored. According to the results, decision-makers should take economic valuation results of informal care into account when considering long-term care-related policies.

## Supplementary information

**Additional file 1.** Questionnaire. The questionnaire employed in the interviews with the informal caregivers

## Data Availability

The original dataset is available from the corresponding author upon request.

## Abbreviations

WTP: Willingness-to-Pay
WTA: Willingness-to-Accept
CVM: Contingent Valuation Method
CCI: Charlson Comorbidity Index
CRA: Caregiver Reaction Assessment
CNY: Chinese Yuan

## References

[1] Lim J, Cowling A (2016). China’s demographic outlook. RBA Bull.

[2] Johnston L, Liu X, Yang M, Zhang X (2016). Getting rich after getting old: China’s demographic and economic transition in dynamic international context. Chin New Sources Econ Growth.

[3] Hu B (2019). Projecting future demand for informal care among older people in China: the road towards a sustainable long-term care system. Health Econ Policy Law.

[4] Qiao N, Ying X, Chen W (2009). A discussion of informal care resource utlization in China. Chin Health Res.

[5] Van den Berg B, Brouwer W, Van Exel J, Koopmanschap M (2005). Economic valuation of informal care: the contingent valuation method applied to informal caregiving. Health Econ.

[6] Michaud P-C, Heitmueller A, Nazarov Z (2010). A dynamic analysis of informal care and employment in England. Labour Econ.

[7] Fast JE, Williamson DL, Keating NC (1999). The hidden costs of informal elder care. J Fam Econ Iss.

[8] Vitaliano PP, Young HM, Zhang J (2004). Is caregiving a risk factor for illness?. Curr Dir Psychol Sci.

[9] Cameron JI, Cheung AM, Streiner DL, Coyte PC, Stewart DE (2006). Stroke survivors’ behavioral and psychologic symptoms are associated with informal caregivers’ experiences of depression. Arch Phys Med Rehabil.

[10] Bugge C, Alexander H, Hagen S (1999). Stroke patients’ informal caregivers: patient, caregiver, and service factors that affect caregiver strain. Stroke.

[11] Bonsang E (2009). Does informal care from children to their elderly parents substitute for formal care in Europe?. J Health Econ.

[12] Geerlings SW, Pot AM, Twisk JW, Deeg DJ (2005). Predicting transitions in the use of informal and professional care by older adults. Ageing Soc.

[13] Oliva-Moreno J, Trapero-Bertran M, Peña-Longobardo LM, del Pozo-Rubio R (2017). The valuation of informal care in cost-of-illness studies: a systematic review. Pharmacoeconomics.

[14] Chiwaula LS, Revill P, Ford D, Nkhata M, Mabugu T, Hakim J (2016). Measuring and valuing informal care for economic evaluation of HIV/AIDS interventions: methods and application in Malawi. Value Health Region Issues.

[15] Koopmanschap MA, Van Exel J, Van den Berg B, Brouwer WB (2008). An overview of methods and applications to value informal care in economic evaluations of healthcare. Pharmacoeconomics.

[16] Van den Berg B, Bleichrodt H, Eeckhoudt L (2005). The economic value of informal care: a study of informal caregivers' and patients' willingness to pay and willingness to accept for informal care. Health Econ.

[17] Lu B, Liu X, Piggott J. Informal long term care in China and population ageing: evidence and policy implications. Popul Rev. 2015;54(2):60–1.

[18] Ouyang P, Sun W, Wang C (2019). Well-being loss in informal care for the elderly people: Empirical study from China national baseline CHARLS. Asia Pac Psychiatry.

[19] Su Q, Peng B (2014). Long-term care of Chinese disabled elderly demand and supply analysis. Soc Sec Stud.

[20] Renyao Z (2011). Analysis on the contradiction between supply and demand of Shanghai’s elderly long-term care services. J Shanghai FinUniv.

[21] Yinghua Z (2019). Pilot and practices of China’s long-term care insurance system.

[22] Hou W, Wang Y, Feng Y, Liu S (2013). Comparison of life satisfaction of informal caregivers and dependent edlerly people. Chin Gerontol.

[23] Joo H, Liang D (2017). Economic burden of informal care attributable to stroke among those aged 65 years or older in China. Int J Stroke.

[24] Carson RT (2012). Contingent valuation: a practical alternative when prices aren't available. J Econ Perspect.

[25] Arrow K, Solow R, Portney PR, Leamer EE, Radner R, Schuman H (1993). Report of the NOAA panel on contingent valuation. Fed Regist.

[26] De Meijer C, Brouwer WB, Koopmanschap M, Van den Berg VEJ (2010). The value of informal care--a further investigation of the feasibility of contingent valuation in informal caregivers. Health Econ.

[27] Garrido-García S, Sánchez-Martínez F, Abellán-Perpiñán JM, Van Exel J (2015). Monetary valuation of informal care based on carers’ and noncarers’ preferences. Value Health.

[28] Whittington D (1998). Administering contingent valuation surveys in developing countries. World Dev.

[29] Anders G, Linus JN, Rupert MS, Mercè B, Anders W, Zbrozek AS (2010). Willingness-to-pay for reductions in care need: estimating the value of informal care in Alzheimer's disease. Int J Geriatric Psychiatry.

[30] Charlson ME, Pompei P, Ales KL, MacKenzie CR (1987). A new method of classifying prognostic comorbidity in longitudinal studies: development and validation. J Chronic Dis.

[31] Van den Berg B, Brouwer WB, Van Exel J, Koopmanschap MA, Van den Bos G, Rutten F (2006). Economic valuation of informal care: lessons from the application of the opportunity costs and proxy good methods. Soc Sci Med.

[32] Given CW, Given B, Stommel M, Collins C, King S, Franklin S (1992). The caregiver reaction assessment (CRA) for caregivers to persons with chronic physical and mental impairments. Res Nurs Health.

[33] Heinze G, Dunkler D (2017). Five myths about variable selection. Transpl Int.

[34] Van den Berg B, Koopmanschap MA (2004). Economic valuation of informal care: an overview of methods and applications. Eur J Health Econ.

[35] Peña-Longobardo LM, Oliva-Moreno J (2015). Economic valuation and determinants of informal care to people with Alzheimer’s disease. Eur J Health Econ.

[36] Joo H, Fang J, Losby JL, Wang G (2015). Cost of informal caregiving for patients with heart failure. Am Heart J.

[37] Brinda EM, Rajkumar AP, Enemark U, Attermann J, Jacob K (2014). Cost and burden of informal caregiving of dependent older people in a rural Indian community. BMC Health Serv Res.

[38] Mentzakis E, Ryan M, McNamee P (2014). Modelling heterogeneity and uncertainty in contingent valuation: an application to the valuation of informal care. Scottish J Political Econ.

[39] Cohen CA, Colantonio A, Vernich L (2002). Positive aspects of caregiving: rounding out the caregiver experience. Int J Geriatric Psychiatry.

[40] Brouwer WB, Van Exel N, Van den Berg B, Van den Bos G, Koopmanschap MA (2005). Process utility from providing informal care: the benefit of caring. Health Policy.

[41] Hanemann WM (1991). Willingness to pay and willingness to accept: how much can they differ?. Am Econ Rev.

[42] Brown TC, Gregory R (1999). Why the WTA–WTP disparity matters. Ecol Econ.

[43] Gervès-Pinquié C, Bellanger MM, Ankri J (2014). Willingness to pay for informal care in France: the value of funding support interventions for caregivers. Heal Econ Rev.

[44] Klose T (1999). The contingent valuation method in health care. Health Policy.

[45] Van den Berg B, Maiwenn AI, Van Exel N, Koopmanschap M, Brouwer W (2008). Economic valuation of informal care: conjoint analysis applied in a heterogeneous population of informal caregivers. Value Health.

[46] Van Exel N, Brouwer WB, Van den Berg B, Koopmanschap MA (2006). With a little help from an anchor: discussion and evidence of anchoring effects in contingent valuation. J Socio Econ.

[47] Landfeldt E, Zethraeus N, Lindgren P (2019). Standardized questionnaire for the measurement, valuation, and estimation of costs of informal care based on the opportunity cost and proxy good method. Appl Health Econ Health Policy.

