# Supplementary material for: Willingness-to-pay and willingness-to-accept of informal caregivers of dependent elderly people in Shanghai, China
Source: BMC Health Serv Res. 2020 Jul 6;20:618. doi: 10.1186/s12913-020-05481-2 (PMC7339502; doi:10.1186/s12913-020-05481-2)
Supplement: Supplementary file 1 — Additional file 1. Questionnaire. The questionnaire employed in the interviews with the informal caregivers [file 12913_2020_5481_MOESM1_ESM.pdf]

Community：______ Questionnaire code：____________ Investigator：___________

Questionnaire

The following questionnaire should be filled by the investigator according to the answers by the interviewee. Please check the boxes below before proceeding with the interview:

□ A brief introduction of the objectives of the study

□ A self-introduction as an investigator

□ The verbal and written consent from the interviewee

□ The absence of the care recipient of the interviewee

Section A — Informal caregiver's information

1. **Gender:** 1. Male □ 2. Female □
2. **Age:** ________
3. **Marital status:** 1. Married □ 2. Widowed □ 3. Divorced □ 4. Unmarried □
4. **Education:** 1. 0-6 years □ 2. 7-15 years □ 3. Above 15 years □
5. **Employment:** 1. Employed □ 2. Retired □ 3. Unemployed □
6. **Monthly income:** 1. Below 4000 □ 2. 4000-6000 □ 3. Above 6000 □
   4. Specific number if provided________
7. **Relationship to the recipient:** 1 The caregiver is the spouse □
   2 The caregiver is the child □
   3 Others, please specify_______
8. **Co-residence with the recipient:** 1. Yes □ 2. No □

Section B — Care recipient's information

9. **Gender:** 1. Male □ 2. Female □
10. **Age:** ________
11. **Education:** 1. 0-6 years □ 2. 7-15 years □ 3. Above 15 years □
12. **Monthly income:** 1. Below 4000 □ 2. 4000-6000 □ 3. Above 6000 □
    4. Specific number if provided________
13. **Morbidities:** 1. Healthy □ 2. Myocardial infarction □
    3. Congestive heart failure □ 4. Peripheral vascular disease □
    5. Cerebrovascular disease □ 6. Dementia □
    7. Chronic pulmonary disease □ 8. Rheumatologic disease □
    9. Peptic ulcer disease □ 10. Mild liver disease □
    11. Diabetes without chronic complication □
    12. Hemiplegia □ 13. Renal disease □

14. Diabetes with chronic complication □
15. Tumor □ 16. Lymphoma □
17. Moderate or severe liver disease □
18. Metastatic solid tumor □ 19 AIDS □
20. Others, please specify: __________________

Section C — Care related information

14. Care duration: _____ months
or since_____ (yyyy) ____ (mm)

15. Care time in last week:

Preparation of food and drinks □	Frequency: ___________ days/week<br>Length of time:___________ h/day
Time investments in shopping, groceries etc. □	Frequency: ___________ days/week<br>Length of time:___________ h/day
Housecleaning □	Frequency: ___________ days/week<br>Length of time:___________ h/day
Washing, ironing or sowing □	Frequency: ___________ days/week<br>Length of time:___________ h/day
Caring for and playing with own children □	Frequency: ___________ days/week<br>Length of time:___________ h/day
Chores, gardening, maintenance □	Frequency: ___________ days/week<br>Length of time:___________ h/day
Aiding patient with personal care □	Frequency: ___________ days/week<br>Length of time:___________ h/day
Aiding patient in visiting the toile □	Frequency: ___________ days/week<br>Length of time:___________ h/day
Aiding patient with indoor movement □	Frequency: ___________ days/week<br>Length of time:___________ h/day
Aiding patient with eating and drinking □	Frequency: ___________ days/week<br>Length of time:___________ h/day
Aiding the patient in travelling outside the building □	Frequency: ___________ days/week<br>Length of time:___________ h/day
Aiding the patient with visiting and in excur sions □	Frequency: ___________ days/week<br>Length of time:___________ h/day
Aiding the patient in contacting health care suppliers □	Frequency: ___________ days/week<br>Length of time:___________ h/day
Aiding patient in organizing home adaptation s, etc. □	Frequency: ___________ days/week<br>Length of time:___________ h/day

Aiding patient in financial matters (insurance, rent, bills) □	Frequency: ___________ days/week<br>Length of time:___________ h/day
Travelling to and from care recipient □	Frequency: ___________ days/week<br>Length of time:___________ h/day
Supervision and companionship □	Frequency: ___________ days/week<br>Length of time:___________ h/day

16. Least preferred task: 1. HDL □ 2. ADL □ 3. IADL □ 4. Supervision and companionship □

17. CRA:

(On a scale from 1 to 5, how does the caregiver agree with the following statements)

I have to stop in the middle of my work or activities to provide care. ____
I have eliminated things from my schedule since caring for the recipient. ____
My activities are centered around care for the recipient. ____
I visit family and friends less because I have been caring for the recipient. ____
The constant interruptions make it difficult to find time for relaxation. ____
I feel privileged to care for the recipient. ____
I really want to care for the recipient. ____
I enjoy caring for the recipient. ____
Caring for the recipient makes me feel good ____
Caring for the recipient is important for me. ____
I will never be able to do enough caregiving to repay the recipient. ____
I resent having to care for the recipient. ____
It is very difficult to get help from my family in taking care of the recipient. ____
Since caring for the recipient, I feel my family has abandoned me. ____
My family left me alone to care for the recipient. ____
My family works together at caring for the recipient. ____
Others have dumped caring for the recipient onto me. ____
It takes all my physical strength to care for the recipient. ____
I am healthy enough to care for the recipient. ____
My health has gotten worse since caring for the recipient. ____
Since caring for the recipient, it seems like I am tired all the time. ____
It is difficult to pay bills. ____
Financial resources are adequate. ____
Caring for the recipient puts a financial strain on me. ____

18. Formal care: 1. Yes, the recipient also uses formal care. □
2. No, the recipient does not receive any formal care. □

19. Other informal Care: 1. Yes, the recipient also receives other informal care. □

2. No, the recipient does not receive any other informal care. □

20. WTP:

Please imagine that the government is to send professional care workers to provide services. What is the maximum amount of money that you are willing to pay for an hour of reduction in caregiving of your least-preferred care task per week?

1. The caregiver provided a specific WTP. □.

___________________ CNY

2. The caregiver refused to reduce any care. □

21. WTA:

Please imagine that the government is going to subsidize the care that you are currently providing to your recipient. How much is the minimum sum that you are willing to accept for this extra hour of your least-preferred care task per week?

1. The caregiver provided a specific WTA. □.

___________________ CNY

2. The caregiver refused to increase any care. □
